# HMGB1 Increases IL-1β Production in Vascular Smooth Muscle Cells via NLRP3 Inflammasome

**DOI:** 10.3389/fphys.2018.00313

**Published:** 2018-03-28

**Authors:** Eun Jung Kim, So Youn Park, Seung Eun Baek, Min A. Jang, Won Suk Lee, Sun Sik Bae, Koanhoi Kim, Chi Dae Kim

**Affiliations:** ^1^Department of Pharmacology, School of Medicine, Pusan National University, Yangsan, South Korea; ^2^Gene and Cell Therapy Research Center for Vessel-associated Diseases, Pusan National University, Yangsan, South Korea

**Keywords:** HMGB1, IL-1β, NLRP3 inflammasome, VSMC, TLRs, RAGE

## Abstract

Vascular smooth muscle cells (VSMCs) are the major cell type in the blood vessel walls, and their phenotypic modulation is a key cellular event driving vascular remodeling. Although high mobility group box-1 (HMGB1) plays a pivotal role in inflammatory processes after vascular injuries, the importance of the links between VSMCs, HMGB1 and vascular inflammation has not been clarified. To prove the hypothesis that VSMCs might be active players in vascular inflammation by secreting inflammatory cytokines, we investigated the proinflammatory effects of HMGB1 and its intermediary signaling pathways in VSMCs. When cultured human VSMCs were stimulated with HMGB1 (10–500 ng/ml), IL-1β production was markedly increased. HMGB1 also increased the expression of NLRP3 inflammasome components including NLRP3, ASC and caspase-1. Among these components, HMGB1-induced expressions of NLRP3 and caspase-1 were markedly attenuated in TLR2 siRNA-transfected cells, whereas ASC and caspase-1 expressions were reduced in RAGE-deficient cells. In TLR4-deficient cells, HMGB1-induced caspase-1 expression was significantly attenuated. Moreover, IL-1β production in HMGB1-stimulated cells was significantly reduced in cells transfected with caspase-1 siRNA as well as in cells treated with monoclonal antibodies or siRNAs for TLR2, TLR4 and RAGE. Overall, this study identified a pivotal role for NLRP3 inflammasome and its receptor signaling involved in the production of IL-1β in VSMCs stimulated with HMGB1. Thus, targeting HMGB1 signaling in VSMCs offers a promising therapeutic strategy for treating vascular remodeling diseases.

## Introduction

Vascular inflammation plays an important role in the pathogenesis of vascular diseases, including vascular remodeling and atherosclerosis (Libby, 2002; Davis et al., 2003). During disease processes, inflammatory mediators are derived from inflammatory cells in vascular lesions, and promote the development of stenotic lesions through the proliferation and migration of vascular smooth muscle cells (VSMCs) (Galis and Khatri, 2002; Waitkus-Edwards et al., 2002). VSMCs are the major cell type in blood vessel walls and play pivotal roles in vascular disease processes by changing phenotype from the contractile to the synthetic phenotype, the latter exhibits distinct proliferative and migratory abilities and produces proinflammatory cytokines (Wang et al., 2012; Ackers-Johnson et al., 2015).

The phenotypic modulation of VSMCs is a key cellular event that drives neointima formation and vascular remodeling. Reportedly, the phenotypic modulation of VSMCs induced by interferon-γ is mediated by high mobility group box-1 (HMGB1), a non-histone chromosomal protein (Wang et al., 2017). HMGB1 is released by monocytes/macrophages in response to inflammatory stimuli and then binds to DNA in a sequence-independent manner and modifies DNA structure, and thereby facilitates gene transcription (Kalinina et al., 2004; Stott et al., 2006; Yang et al., 2015). In blood vessels, high levels of extracellular HMGB1 have been detected in human atherosclerotic plaque, and reportedly are implicated in vascular inflammation by potentiating inflammatory responses (Kalinina et al., 2004; Cai et al., 2015). It has also been shown that HMGB1 modulates the phenotype of VSMCs toward the activated synthetic phenotype and stimulates MCP-1/CCL2 gene expression through toll-like receptor 4 (TLR4) (Cai et al., 2015; Wang et al., 2017). However, the nature of the links between VSMCs, HMGB1, and vascular inflammation have not been clarified.

Inflammasomes are key regulators of HMGB1-induced inflammation (Chi et al., 2015). These are cytoplasmic, high-molecular weight, multisubunit protein complexes capable of inducing inflammatory response by releasing IL-1β (Guo et al., 2015; Lamkanfi and Dixit, 2015), which is a pivotal player in vascular inflammatory processes (Kirii et al., 2003; Chamberlain et al., 2006). Inflammasomes are composed of an inflammasome sensor molecule, the adaptor protein ASC (apoptosis-associated speck-like protein), and procaspase-1 (Martinon et al., 2002; Chamberlain et al., 2006; Guo et al., 2015). Following activation, the inflammasome complex induces autocatalytic cleavage of procaspase-1 into its active form, which can cleave pro-IL-1β into its mature and released forms (Netea and Joosten, 2015). Thus, it is proposed that HMGB1 contributes to vascular inflammation by promoting the activation of NLRP3 (NLR family pyrin domain containing proteins 3) inflammasome and the processing of IL-1β in vascular cells.

Cell membrane pattern recognition receptors (PRRs), including TLR2, TLR4, and RAGE (receptor for advanced glycation end products), interact with HMGB1, and then mediate the production of pleiotropic cytokines in inflammatory cells (Park et al., 2003, 2006; Treutiger et al., 2003). In monocytes/macrophages, HMGB-1 binding to TLR4 enhanced inflammatory response through the synthesis of IL-1β (Andersson et al., 2002; Yang et al., 2010). which plays an essential role in the complex inflammatory process. Previous studies have also reported that TLR2 and TLR4 can activate NLRP3 inflammasome, and thus facilitate the secretions of inflammatory cytokines (Qi et al., 2014; Koch and Müller, 2015), which indicates all three PRRs may mediate HMGB1-induced inflammation. Although it was suggested that HMGB1 secretion by activated VSMCs seems to be critically involved in vascular inflammation, its role in the production of inflammatory cytokines in human VSMCs is not well clarified.

Inflammasomes are molecular platforms that trigger the maturation of proinflammatory cytokines in inflammatory cells when cells are exposed to stress. In the injured vasculatures, HMGB1 plays a pivotal role in the process of vascular inflammation. However, the links between HMGB1 and inflammasome in VSMCs has not been clarified. To prove the hypothesis that VSMCs might be active players in vascular inflammation by secreting inflammatory cytokines, we investigated the proinflammatory effects of HMGB1 and its intermediary signaling pathways in human VSMCs.

## Materials and methods

### Chemicals and antibodies

Recombinant human high mobility group box 1 (HMGB1), anti-NLRP3, and anti-IgG antibodies were purchased from R&D Systems, Inc. (Minneapolis, MN). NLRP3-inflammasome inhibitor, MCC-950 was purchased from Invivogen (San Diego, CA). Anti-IL-1β, anti-TLR2, anti-TLR4, anti-RAGE, and anti-caspase-1 antibodies were from Abcam (Cambridge, MA). β-Actin antibody was purchased from Santa Cruz Biotechnology Inc. (Beverly, MA), and anti-ASC antibody from Adipogen (San Diego, CA).

### Cell culture

Human aortic smooth muscle cells were purchased from the ATCC (Manassas, VA). Cells were grown in culture dishes using smooth muscle cell growth medium (Gibco BRL, Grand Island, NY), smooth muscle growth supplement (Gibco BRL), and 10% fetal bovine serum (FBS), antibiotic antimycotic (Gibco BRL).

### RNA isolation and RT-PCR

IL-1β and NLRP3 mRNA expressions in VSMCs were quantified by RT-PCR using GAPDH mRNA as an internal standard. Total RNA was isolated using Trizol reagent (Invitrogen, Carlsbad, NY), according to the manufacturer's instructions. RNA (1 μg) were reverse transcribed into cDNA using the ImProm-II reverse transcription system (Promega, Madison, WI). PCR amplification was performed using the IL-1β specific primers (forward, 5′-GGG CCT CAA GGA AAA GAA TC-3′; reverse, 5′-TTC TGC TTG AGA GGT GCT GA-3′) and the NLRP3 specific primers (forward, 5′-GCG CCT CAG TTA GAG GAT GT-3′; reverse, 5′-ACC AGC TAC AAA AAG CAT GGA-3′). Equal amounts of RT-PCR products were separated on 1% agarose gels and stained with ethidium bromide. Signals from bands were quantified using the Image J densitometry program, and data were expressed as relative GAPDH densities.

### Western blot assay

Cells lysates were prepared in lysis buffer (Thermo Scientific, Rockford, IL), and equal amounts of the proteins obtained were separated on 10~15% polyacrylamide gels under reducing conditions, and then transferred to nitrocellulose membranes (Amersham-Pharmacia Biotech, Piscataway, NJ). Membranes were blocked with 5% skim milk in tris buffered saline Tween-20 (TBST), incubated overnight with primary antibody in 5% skim milk, washed with TBST, and incubated with HRP-conjugated secondary antibody for 2 h. Blots were developed using enhanced chemiluminescence (ECL) Western blot detection reagents (Amersham-Pharmacia Biotech).

### Small interfering RNA (siRNA) preparation and transfection

TLR2 (GenBank accession no. NM_003264.3), TLR4 (GenBank accession no. NM_1385554.2), RAGE (GenBank accession no. NM_001136.3), and caspase-1 (GenBank accession no. NM_021571.2) siRNA oligonucleotides were synthesized by Bioneer (Daejeon, Korea). The siRNA negative control duplex was used as a control oligonucleotide. siRNA molecules were transfected into cells using Lipofectamine 2000 (Invitrogen) according to the manufacturer's instructions.

### Enzyme-linked immunosorbent assay

Levels of IL-1β in the culture media were measured using ELISA kits (Affymetrix, Inc., Santa Clara, CA) according to the manufacturer's instructions.

### Statistical analysis

Results were expressed as means ± SEMs. The analysis was conducted using one-way analysis of variance (ANOVA) followed Dunnett Multiple Comparison Test. The Student's *t*-test was used to determine the significances of treatment effects. Statistical significance was accepted for *p*-values < 0.05

## Results

### HMGB1 increased IL-1β production in human vascular smooth muscle cells

To assess the effect of HMGB1 on IL-1β production in cultured human VSMCs, cells were serum starved for 24 h, and then stimulated with recombinant HMGB1 (HMGB1) at various concentrations. RT-PCR and Western blot analyses indicated that IL-1β mRNA and protein were expressed at low levels in VSMCs in the absence of HMGB1 stimulation, but when VSMCs were stimulated with 10–500 ng/ml of HMGB1, both IL-1β mRNA expression and protein production were markedly increased. In this experiment, dose-dependency was observed up to HMGB1 concentration of 100 ng/ml, and thus, HMGB1 was used at this concentration in subsequent experiments.

**Figure 1:**
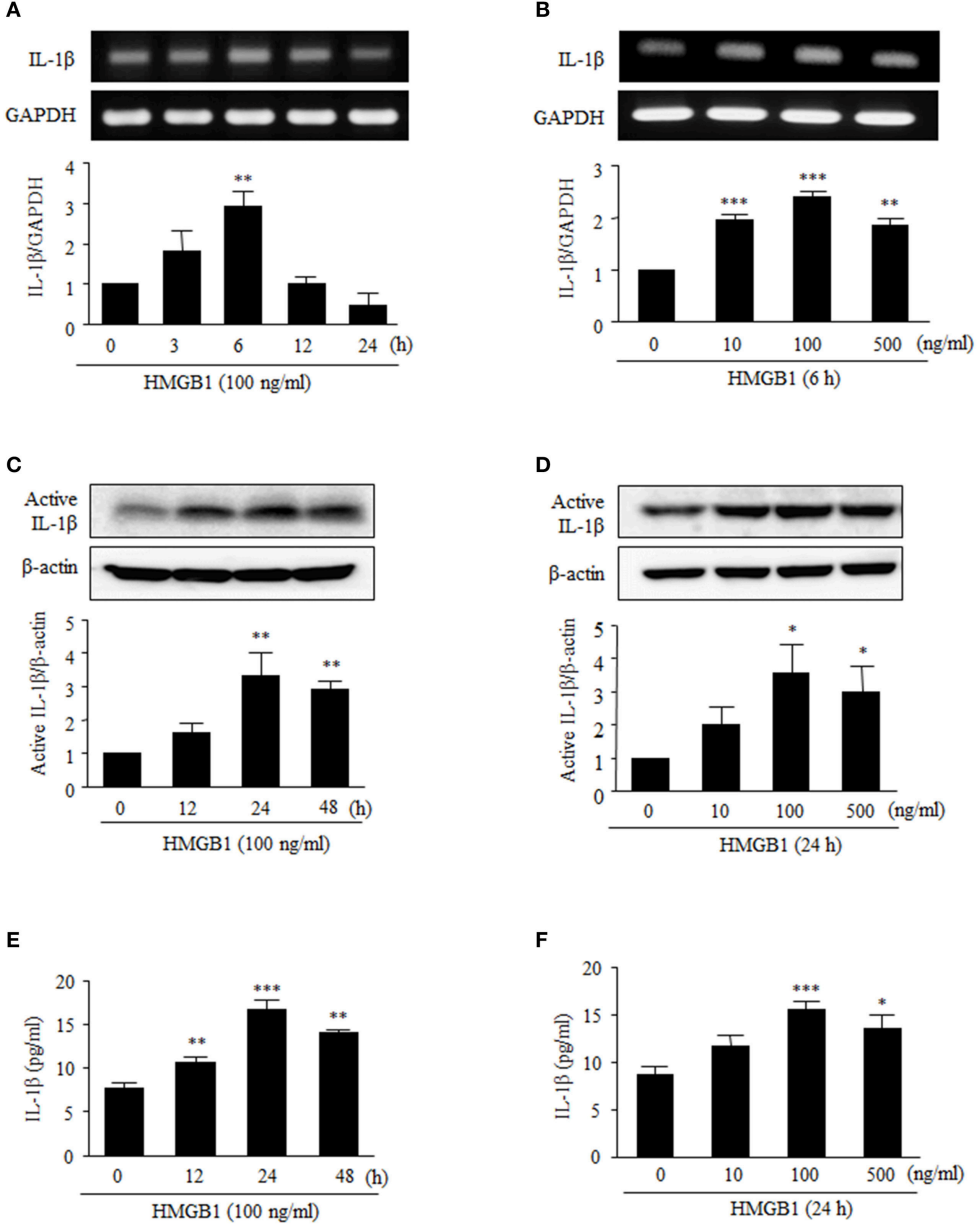
Effects of HMGB1 on IL-1β expression and its release from VSMCs. VSMCs were treated with HMGB1 (100 ng/ml) for 0–24 h, and were also treated with HMGB1 (0–500 ng/ml) for 6 h. **(A,B)** The mRNA levels of IL-1β were determined by RT-PCR. GAPDH was used as a control. Data are expressed as means ± SEMs of duplicates pooled from 4 independent experiments. **(C,D)** The protein levels of active IL-1β were determined by Western blot. β-Actin expression served as an internal control. Data are expressed as means ± SEMs of duplicates pooled from 4 independent experiments. **(E,F)** VSMCs were treated with HMGB1 (100 ng/ml) for 0–48 h, and were also treated with HMGB1 (0–500 ng/ml) for 24 h. The levels of IL-1β in the culture media were quantified by ELISA. Data are expressed as means ± SEMs of triplicates pooled from 4 independent experiments. ^*^P < 0.05, ^**^P < 0.01, and ^***^P < 0.001 vs. control (untreated cells).

As shown in Figures 1A,B, RT-PCR analysis showed that IL-1β mRNA levels after treatment with HMGB1 (100 ng/ml, 6 h) significantly increased by 2.88 ± 0.25-fold (*P* < 0.01). We next analyzed the subsequent cleavage and secretion of active IL-1β, as VSMCs can be situationally an immune effector cells. When VSMCs were stimulated with HMGB1 (100 ng/ml), levels of active IL-1β protein significantly increased up to 24 h (Figures 1C,D). Similarly, levels of IL-1β in culture medium were significantly increased at 12–48 h and peaked at 24 h (Figures 1E,F).

### HMGB1 increased the expression of NLRP3 inflammasome components

NLRP3-dependent inflammasome is multiprotein complex, consisting of NLRP3, ASC and caspase-1, which processes proIL-1β into IL-1β (Dinarello, 2009). We then examined whether HMGB1 could affect the expression of the NLRP3 inflammasome in human VSMCs.

**Figure 2:**
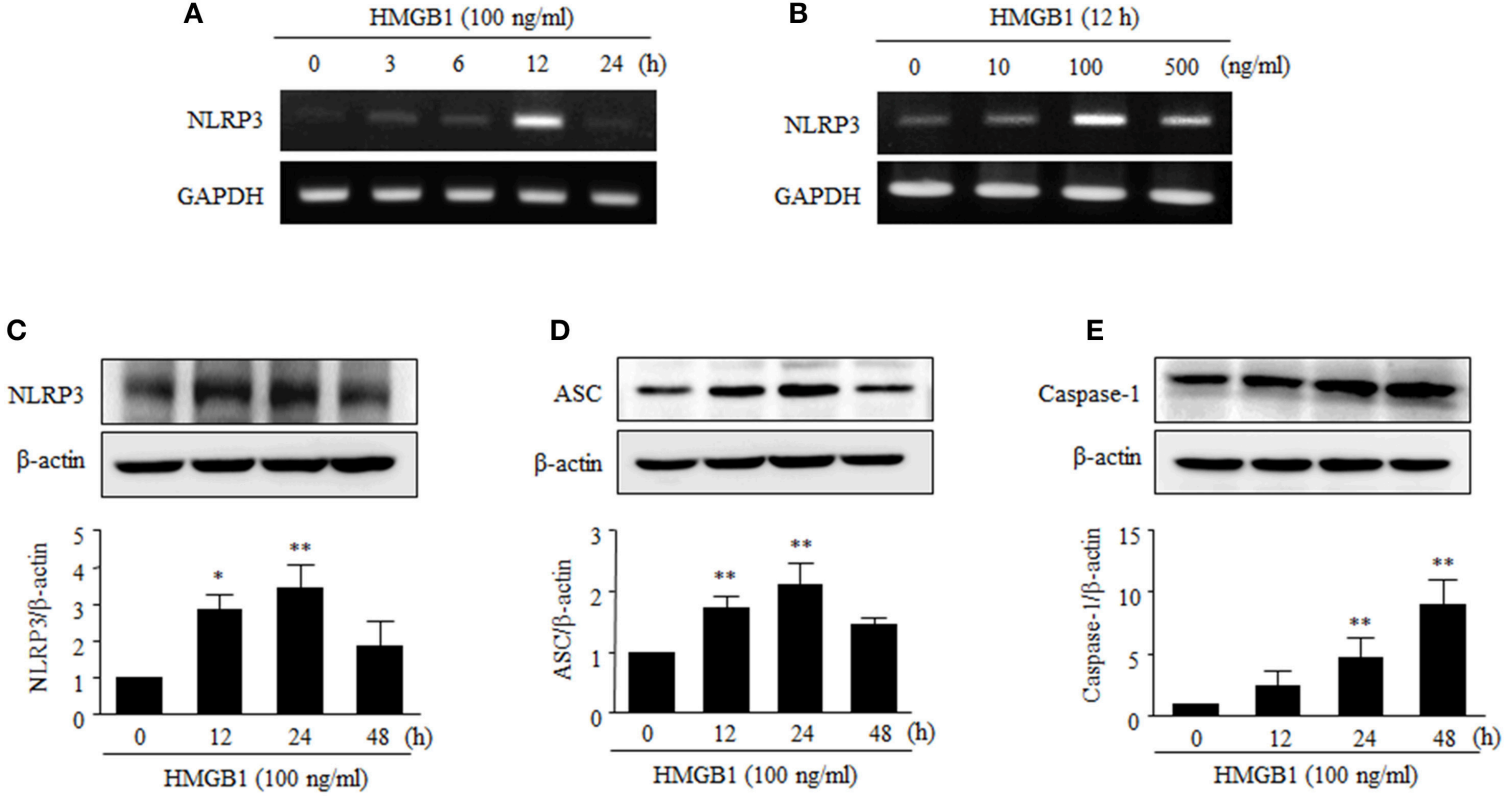
Effects of HMGB1 on the expression of NLRP3 inflammasome in VSMCs. **(A,B)** VSMCs were treated with HMGB1 (100 ng/ml) for 0–24 h, and also treated with HMGB1 (0–500 ng/ml) for 12 h. The mRNA levels of NLRP3 were determined by RT-PCR using GAPDH as a control. **(C–E)** VSMCs were treated with HMGB1 (100 ng/ml) for 0–48 h. The protein levels NLRP3, ASC, and Caspase-1 were determined by Western blot using β-actin as an internal control. Data are expressed as means ± SEMs of duplicates pooled from 4 independent experiments. ^*^P < 0.05 and ^**^P < 0.01 vs. control (untreated cells).

NLRP3 mRNA levels peaked after 12 h of stimulation with HMGB1 (100 ng/ml), and this induction was also observed when cells were stimulated with HMGB1 at various concentrations (0–500 ng/ml) (Figures 2A,B). Furthermore, stimulation of human VSMCs with HMGB1 (100 ng/ml) markedly increased NLRP3 (3.43 ± 0.62-fold, *P* < 0.01) and ASC (2.47 ± 0.34-fold, *P* < 0.01) at 24 h and caspase-1 (8.95 ± 2.01-fold, *P* < 0.01) at 48 h, respectively (Figures 2C–E).

### Dependence of HMGB1-induced NLRP3 inflammasome on TLR2, TLR4, and RAGE

It was reported that extracellular HMGB1 stimulates inflammatory cells by activating its receptors, including TLR2, TLR4, and RAGE that are involved in passive process of inflammation (Mitola et al., 2006). In the present study, TLR2, TLR4, and RAGE were found to be constitutively expressed on cultured human VSMCs (data not shown).

**Figure 3:**
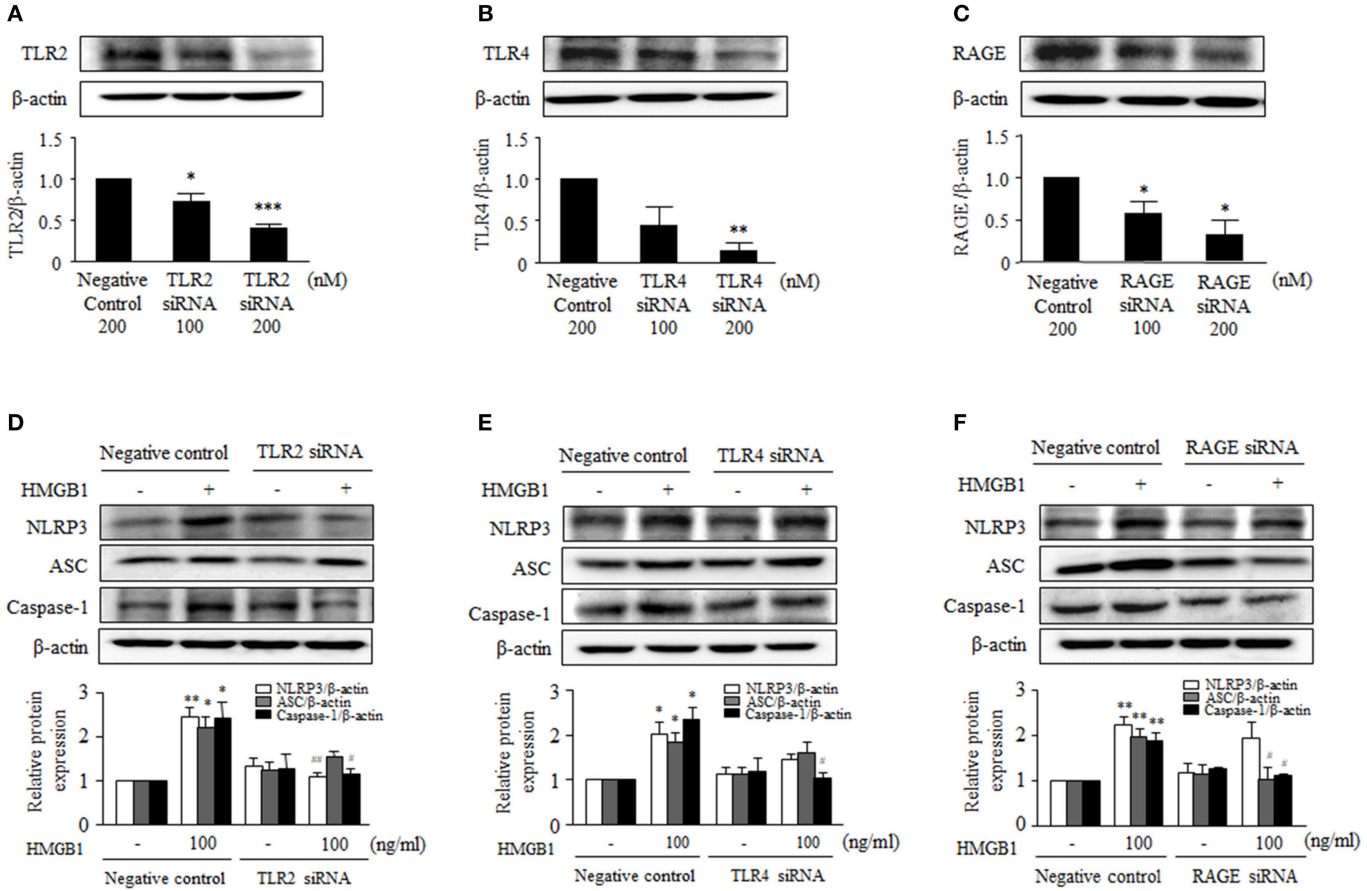
Involvement of TLR2, TLR4, and RAGE in HMGB1-induced NLRP3 inflammasome expression in VSMCs. **(A–C)** VSMCs were transfected with siRNA for TLR2, TLR4, and RAGE, and knockdown efficacies were determined by Western blot using β-actin as an internal control. Data are expressed as means ± SEMs of duplicates pooled from 3 independent experiments. ^*^P < 0.05, ^**^P < 0.01, and ^***^P < 0.001 vs. control (untreated cells). **(D–F)** VSMCs were transfected with siRNA (200 nM) for TLR2, TLR4, and RAGE, and then stimulated with HMGB1 for 24 h. The expressions of NLRP3, ASC, and Caspase-1 were determined by Western blot using β-actin as an internal control. Data are expressed as means ± SEMs of duplicates pooled from 4 to 6 independent experiments. ^*^P < 0.05, ^**^P < 0.01 vs. corresponding value in untreated cells. ^#^*P* < 0.05 and ^##^*P* < 0.01 vs. corresponding value in negative control.

To identify the receptors that mediate NLRP3 inflammasome expression in HMGB1-stimulatd VSMCs, the expression of inflammasome components was determined in cells transfected with siRNAs for TLR2, TLR4, or RAGE. The transfection of target receptor-specific siRNAs (200 nM) reduced the protein expression of TLR2, TLR4, and RAGE to ~59, ~86, and ~67% of the control level, respectively (Figures 3A–C). In cells transfected with scrambled siRNA duplex (negative controls), HMGB1 (100 ng/ml) significantly elevated the protein levels of NLRP3 (2.45 ± 0.20-fold, *P* < 0.01), ASC (2.19 ± 0.25-fold, *P* < 0.05) and caspase-1 (2.42 ± 0.35-fold, *P* < 0.05) (Figures 3D–F). TLR2 gene-knockdown cells exhibited significantly less protein levels of NLRP3 (1.10 ± 0.09-fold, *P* < 0.01) and caspase-1 (1.15 ± 0.13-fold, *P* < 0.05) in response to HMGB1 (100 ng/ml), while the protein levels of ASC (1.55 ± 0.13-fold) showed marginal effect (Figure 3D). TLR4 gene-knockdown cells showed a significant attenuation only in the caspase-1 protein expression increased by HMGB1 (1.03 ± 0.21-fold, *P* < 0.05; Figure 3E). In cells subjected to RAGE gene-knockdown, the expressions of ASC and caspase-1 protein were similarly decreased in response to HMGB1, while the expression of NLRP3 protein was not affected by RAGE gene-knockdown (Figure 3F).

### Dependences of HMGB1-induced IL-1β production on TLR2, TLR4, and RAGE

**Figure 4:**
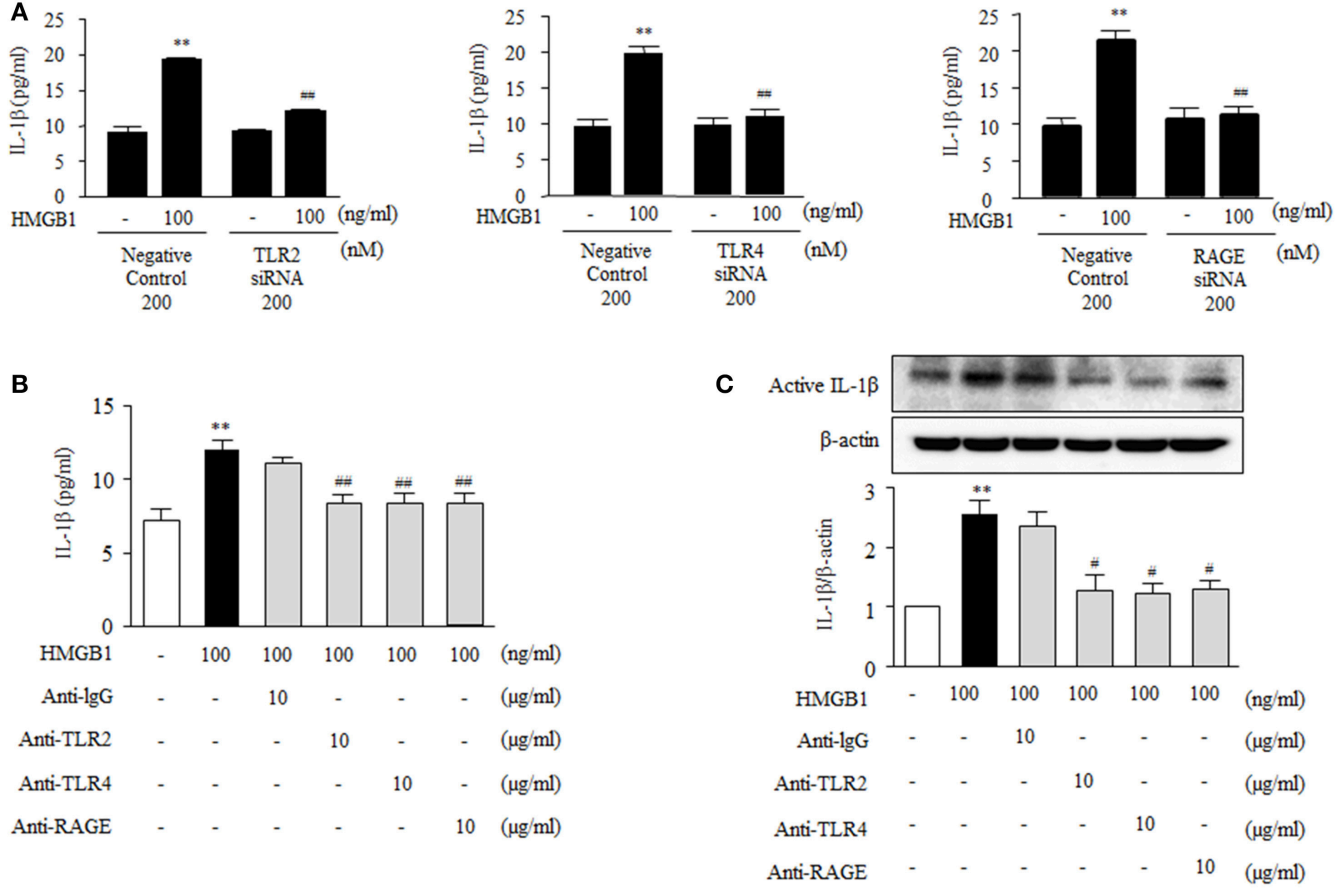
Functional role of HMGB1 receptors on the production of IL-1β in HMGB1-stimulated VSMCs. **(A)** VSMCs were transfected with siRNA (200 nM) for TLR2, TLR4, and RAGE, and then incubated with HMGB1 for 24 h. The levels of IL-1β in the culture media were quantified by ELISA. Data are expressed as means ± SEMs of triplicates pooled from 4 independent experiments. ^**^P < 0.01 vs. non-treated control. ^##^*P* < 0.01 vs. corresponding value in negative control. **(B)** VSMCs were pretreated with anti-IgG antibody (10 μg/ml), anti-TLR2 antibody (10 μg/ml), anti-TLR4 antibody (10 μg/ml), or anti-RAGE antibody (10 μg/ml) for 30 min, and then stimulated with HMGB1 (100 ng/ml) for 24 h. IL-1β release into culture media was quantified by ELISA. Data are expressed as means ± SEMs of triplicates pooled from 4 independent experiments. **(C)** Protein levels of active IL-1β were assessed by Western blot using β-actin as an internal control. Data are expressed as means ± SEMs of duplicates pooled from 4 independent experiments. ^**^P < 0.01 vs. non-treated control. ^#^*P* < 0.05 and ^##^*P* < 0.01 vs. control in HMGB1-treated cells.

To further investigate the functional role of HMGB1 receptors on the production of inflammatory cytokines in VSMC, we measured IL-1β produced in cells deficient of TLR2, TLR4 and RAGE. As shown in Figure 4A, IL-1β production was markedly increased in HMGB1 (100 ng/ml)-stimulated VSMCs transfected with negative controls (19.94 ± 0.34 pg/ml, *P* < 0.01), which was significantly attenuated in cells transfected with siRNAs for TLR2, TLR4, and RAGE. In cells pretreated with neutralizing antibodies, as was expected, IL-1β induction by HMGB1 was significantly inhibited by anti-TLR2 antibody (8.36 ± 0.61-fold, *P* < 0.01), anti-TLR4 antibody (8.34 ± 0.69-fold, *P* < 0.01) and anti-RAGE antibody (8.38 ± 0.67-fold, *P* < 0.01), whereas anti-IgG antibody had no effect (Figure 4B). In addition, Western blot analysis showed that the extent of induction of active IL-1β protein by HMGB1 (100 ng/ml) was significantly reduced by pretreating cells with anti-TLR2 antibody, anti-TLR4 antibody or anti-RAGE antibody (Figure 4C). These results suggest that HMGB1 increases the production of IL-1β via NLRP3 inflammasome activated by TLR2, TLR4, and RAGE signaling pathways.

### HMGB1 increases the production of IL-1β via activation of NLRP3 inflammasome

**Figure 5:**
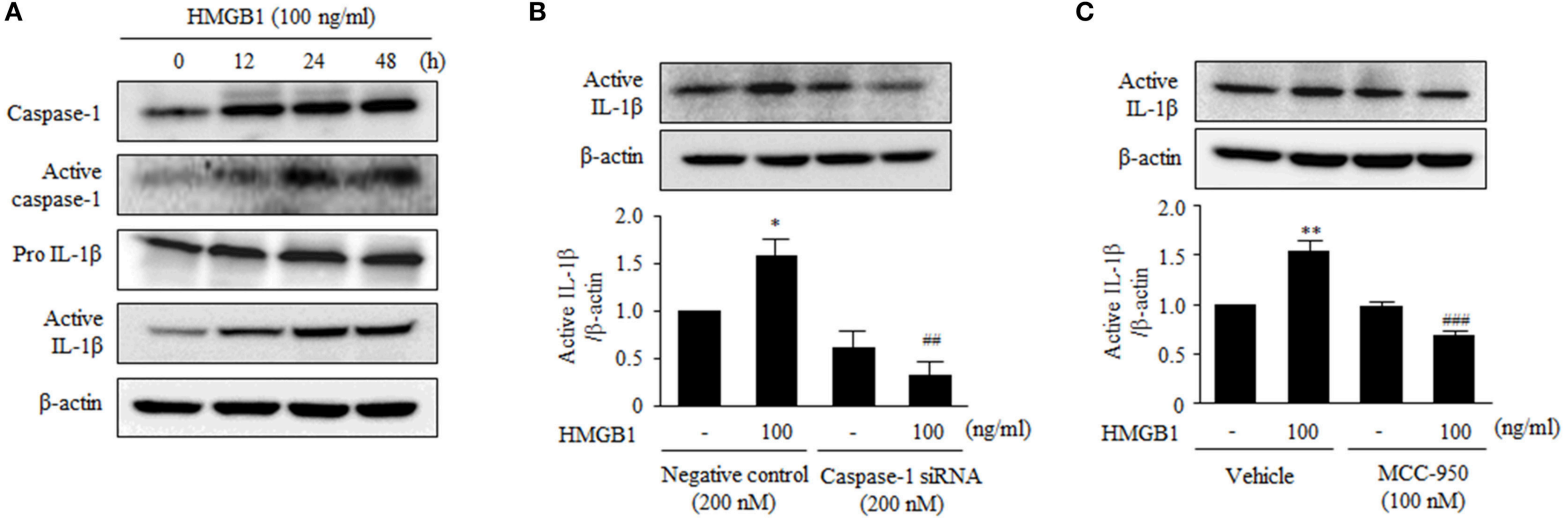
Functional role of NLRP3 inflammasome on the production of IL-1β in HMGB1-stimulated VSMCs. **(A)** VSMCs were treated with HMGB1 (100 ng/ml) for 0–48 h. The protein levels of caspase-1, active caspase-1 (p20), pro- IL-1β, and active IL-1β were assessed by Western blot using β-actin as an internal control. **(B)** VSMCs were transfected with caspase-1 siRNA (200 nM), and then stimulated with HMGB1 for 24 h. The expression of active IL-1β was determined by Western blot using β-actin as an internal control. Data are expressed as means ± SEMs of duplicates pooled from 4 independent experiments. ^*^P < 0.05 vs. non-treated control. ^##^*P* < 0.01 vs. corresponding value in negative control. **(C)** VSMCs were pretreated with MCC-950 (100 nM; a NLRP3 inhibitor) for 30 min and then stimulated with HMGB1 for 24 h. The protein levels of active IL-1β were determined by Western blot using β-actin as an internal control. Data are expressed as means ± SEMs of duplicates pooled from 4 independent experiments. ^**^P < 0.01 vs. non-treated control in vehicle. ^###^*P* < 0.001 vs. corresponding value in HMGB1-treated cells.

The stimulatory effect of HMGB1 on the expression of NLRP3 components in human VSMCs prompted us to investigate the effect of HMGB1 on the production of IL-1β. To determine the activity of NLRP3 inflammasome in HMGB1-stimulated cells, we measured cleaved caspase-1 (p20) protein levels in this study. As shown in Figure 5A, stimulation of VSMCs with HMGB1 (100 ng/ml) increased the activity of caspase-1, as determined by cleaved caspase-1 (p20) levels. Likewise, the production of active IL-1β was markedly increased in cells stimulated with HMGB1.

To further determine the role of caspase-1 on the production of inflammatory cytokines, we measured IL-1β production in VSMCs transfected with caspase-1 siRNA. Transfection of VSMCs with caspase-1 siRNA (200 nM) reduced the expression of caspase-1 to ~42% of the control level (data not shown). In caspase-1-deficient cells, active IL-1β was not increased in response to HMGB1 (100 ng/ml), whereas it was significantly increased in HMGB1-stimulated cells transfected with negative control (Figure 5B). In addition, the production of active IL-1β protein induced by HMBG1 was significantly suppressed when cells were pretreated with MCC-950, a selective inhibitor of the NLRP3 inflammasome (Figure 5C).

## Discussion

Vascular injury initiates inflammatory cell infiltration into damaged tissues, and this is followed by increased production of inflammatory cytokines, which can cause vascular remodeling diseases (Eid et al., 2009; Li et al., 2012; Song et al., 2012). Previous studies have argued that IL-1β plays a crucial regulatory role in vascular inflammation, but little is known of the role played by VSMCs in damaged vasculatures. Here, we provide direct evidence that stimulation of human VSMCs with HMGB1 induced the release of IL-1β in association with an increased expression of NLRP3 inflammasome components, including NLRP3, ASC and caspase-1. In addition, HMGB1-induced IL-1β production by VSMCs was attenuated by inhibiting TLR2, TRL4 and RAGE signaling pathways or by inhibition of NLRP3 inflammasome. These observations suggest that VSMCs actively participate in vascular inflammatory processes by secreting inflammatory cytokines.

HMGB1 is one of the best characterized damage-associated molecular pattern, and activates NLRP3 inflammasome and NF-κB in inflammatory cells (Pisetsky et al., 2008; Chi et al., 2015). NLRP3 inflammasome is composed of NLRP3 protein, the adaptor molecule ASC, which contains two death-fold domains (one pyrin domain and one CARD), and pro-caspase-1 (Sutterwala et al., 2006; Chae et al., 2011). NLRP3 inflammasome can be activated by a variety of stimuli, such as extracellular ATP released by dying cells (Mariathasan et al., 2006), the phospholipid cardiolipin, mitochondrial DNA (Nakahira et al., 2011; Iyer et al., 2013), and bacterial toxins (Muñoz-Planillo et al., 2009). NLRP3 inflammasome activation requires two steps: priming and the inflammasome complex assembly (Sutterwala et al., 2014). The priming step is initiated by pattern recognition receptors, cytokine receptors, or any factor able to induce the activation of NF-κB, and these initiations result in the upregulation of NLRP3 to a functional level and pro-IL-1β expression (Sutterwala et al., 2014). The second step is posttranscriptional and enables the assembly of NLRP3 inflammasome complex (Bauernfeind et al., 2009; Sutterwala et al., 2014). In the present study, stimulation of VSMCs with HMGB1 upregulated the mRNA and protein expressions of NLRP3 components, including NLRP3, ASC and caspase-1, which suggests HMGB1 can initiate the activation of inflammasome in VSMCs. However, it remains to be determined whether the assembly of NLRP3 inflammasome complex in VSMCs requires the presence of HMGB1.

HMGB1 primarily resides in the nuclei of quiescent cells, when cells are exposed to stress, HMGB1 can be translocated into the extracellular milieu, where it elicits the production of proinflammatory mediators and induces the infiltration of inflammatory cells (Harris et al., 2012; Yang et al., 2013). HMGB1 has been implicated in the pathogenesis of a variety of inflammatory diseases, and under these pathological conditions, the levels of HMGB1 are elevated in tissues and serum associated with the development of inflammation (Kalinina et al., 2004; Andrassy et al., 2008). Studies have showed that high levels of extracellular HMGB1 in atherosclerotic plaque were found in human blood vessels, and suggested its involvement in vascular inflammation via the potentiation of inflammatory processes (Kalinina et al., 2004; Cai et al., 2015). In addition to macrophages and endothelial cells, VSMCs have been identified as a major source of HMGB1 production in atherosclerotic lesions, and recent evidence suggests HMGB1 is required for the development of vascular inflammation and neointimal lesions following vascular injury (Chen et al., 2012; Zou et al., 2014). Furthermore, HMGB1 was reported to modulate the phenotype of VSMC toward activated synthetic type (Cai et al., 2015; Wang et al., 2017). Therefore, HMGB1 secretion by activated VSMCs might be critically involved in the process of vascular inflammation, and suggested as a potential therapeutic target to prevent vascular remodeling diseases.

Recently, it was suggested that VSMCs might be an active player in vascular inflammatory processes by secreting inflammatory cytokines (Wang et al., 2012; Ackers-Johnson et al., 2015). In a previous study, IL-1β was found to play an essential role in the complex inflammatory process by modulating the expression of genes induced by the transcription factors AP-1 and NF-κB, and also enhance VSMC proliferation and migration via P2Y2 receptor-mediated RAGE expression and HMGB1 release (Eun et al., 2015). Furthermore, it has been reported that arterial neointima formation and atherosclerotic lesion areas were lower in IL-1β knockout mice than in controls (Kirii et al., 2003; Chamberlain et al., 2006), suggesting IL-1β as a pivotal player in vascular inflammatory processes. Based on these reports and our present data in which stimulation of VSMC with HMGB1 increased NLRP3 inflammasome components in association with an increase in IL-1β production, it was suggested that the HMGB1-stimulated VSMCs actively induce vascular inflammation by producing inflammatory cytokines.

The proinflammatory function of HMGB1 relies on its binding to certain cell membrane pattern recognition receptors (PRRs), including RAGE, TLR2, and TLR4 (van de Veerdonk et al., 2011). Previous studies have reported that the activations of RAGE, TLR2, or TLR4 can increase the expression of proinflammatory cytokines in different cell types (Szomolanyi-Tsuda et al., 2006; He et al., 2012; Rhee et al., 2013). Moreover, TLR2 and TLR4 can also activate NLRP3 inflammasomes, which then facilitate the maturation and secretion of inflammatory cytokines (Qi et al., 2014; Koch and Müller, 2015). Thus, all three PRRs were suggested as mediators involved in the HMGB1-induced production of inflammatory cytokines in keratinocytes. In line with these results, this study also showed the constitutive expression of HMGB1-binding PRRs including RAGE, TLR2, and TLR4 in cultured human VSMCs. Interestingly, the HMGB1-induced expression of NLRP3 and caspase-1 was markedly attenuated in TLR2-deficient cells, whereas ASC and caspase-1 expression was inhibited in RAGE-deficient cells. In TLR4-deficient cells, HMGB1-induced caspase-1 expression was markedly attenuated. Although the expression of inflammasome components was differentially affected by inhibition of various PRRs, caspase-1 expression induced in HMGB1-stimulated cells was markedly reduced in all cells transfected with siRNAs for TLR2, TLR4 and RAGE. Thus, caspase-1 was considered as a common component that mediates PRRs signals in HMGB1-stimulated VSMCs. On the basis of the importance of caspase-1 in the activation of inflammasome and the experimental results of this study in which IL-1β production in HMGB1-stimulated cells was markedly reduced in cells transfected with caspase-1 siRNA, it is possible that IL-1β production in HMGB1-stimulated cells might be similarly reduced among cells treated with various inhibitors for TLR2, TLR4, and RAGE.

In conclusion, the principal finding made in this study was that HMGB1 induced IL-1β production in VSMCs via the increased expression of NLRP3 inflammasome components with subsequent activation of caspase-1. Moreover, this study identified a novel receptor signaling pathway involved in the expression of inflammasome components in VSMCs stimulated with HMGB1. Overall, this study provides new insights of innate responses that contribute to the pathogenesis of vascular inflammation, thus, targeting HMGB1 signaling in VSMCs offers a promising therapeutic strategy for treating vascular remodeling diseases.

## Author contributions

EK and SP designed and performed experiments, analyzed the experimental data, and wrote the manuscript. CK contributed to design and the writing. MJ and SEB performed experiments. WL, SSB, and KK approved manuscript.

### Conflict of interest statement

The authors declare that the research was conducted in the absence of any commercial or financial relationships that could be construed as a potential conflict of interest.
